# Fingerprints of volatile flavor compounds from southern stinky tofu brine with headspace solid‐phase microextraction/gas chromatography–mass spectrometry and chemometric methods

**DOI:** 10.1002/fsn3.943

**Published:** 2019-01-30

**Authors:** Pao Li, Jing Xie, Hui Tang, Cong Shi, Yanhua Xie, Jing He, Yulun Zeng, Hongli Zhou, Bo Xia, Chunyan Zhang, Liwen Jiang

**Affiliations:** ^1^ Hunan Provincial Key Laboratory of Food Science and Biotechnology College of Food Science and Technology Hunan Agricultural University Changsha China; ^2^ Hunan Agricultural Product Processing Institute Hunan Academy of Agricultural Sciences Changsha China

**Keywords:** chemometric methods, fingerprints, organic volatile flavor compounds, solid‐phase microextraction, southern stinky tofu

## Abstract

It is difficult to produce southern stinky tofu, a famous traditional Chinese snack, at industry scale due to the complex composition of its brine. In this study, the fingerprints of organic volatile flavor compounds in the southern stinky tofu brine samples from five manufacturers were studied using headspace solid‐phase microextraction/gas chromatography–mass spectrometry (HS‐SPME/GC‐MS) with the aid of chemometric methods. The fingerprints were obtained by HS‐SPME/GC‐MS and analyzed with the time shift alignment method, Shannon entropy, correlation coefficient, and principal component analysis. The results show that the time shifts in the samples can be accurately corrected by the time shift alignment method despite unexpected interferences. The fingerprint information was evaluated by Shannon entropy, while the similarities and differences in the fingerprints were investigated by correlation coefficient. Moreover, the identification of stinky tofu manufacturers can be achieved by principal component analysis. The predominant volatile compounds in southern stinky tofu brines were indole, 3‐methylindole, phenol, and 4‐methylphenol. Therefore, the established fingerprinting of volatile compounds for the brines by combining HS‐SPME/GC‐MS with chemometric methods was a simple and reliable method.

## 1 INTRODUCTION

Southern stinky tofu, a kind of well‐known traditional Chinese soybean food which smells smelly but tastes tasty, is also called Chinese cheese (Liu, Han, & Zhou, 2011; Liu, Miao, Wei, & Sun, 2012). The southern stinky tofu is unfermented and made from tofu cubes soaked in special stinky brine. The quality of southern stinky tofu mainly depends on the quality of the brine, which is made from various fermented ingredients, such as bamboo shoots, fish, and shrimps, producing a strong stinky odor (Xie, Lin, & Jiang, 2015). However, without accurate quantitative analytical technology, it is difficult for stinky tofu brine to be industrialized and commercialized. The crucial process parameters of brine manufacturing are not yet identified, making it difficult to satisfy batch repeatability and to scale up in food industry (Xu & Jiang, 2014).

Different odor characteristics of the brine samples from different fermentation periods and manufacturers can be observed, and the research of the fingerprints of volatile flavor compounds in the brine samples helps to optimize quality control of these products. As an advanced analytical technique to analyze flavor compounds in food samples, headspace solid‐phase microextraction/gas chromatography–mass spectrometry (HS‐SPME/GC‐MS) has many advantages, such as easy to perform, solvent free, sensitive, and selective (Canellas, Vera, & Nerín, 2016; Lv et al., 2017; Wang et al., 2017; Xiao et al., 2017; Xu & Jiang, 2014). However, the use of this method in the analysis of organic volatile flavor compounds in southern stinky tofu brine has been very limited, because the research of organic volatile flavor compounds is a very difficult task (Chao, Tomii, Watanabe, & Tsai, 2008; Liu, Chen, Sun, & Huang, 2009). The evaluation of these compounds in the complex sample is a challenging to GC methods due to the overlapping signals and the high number of compounds. Besides, many factors can influence the chemical composition, including different raw materials and process parameters. Different instruments or conditions for any particular product may also lead to differences between samples of the same product (Zeng, Liang, & Xu, 2005). Furthermore, it is a difficult task to obtain information about the presence or absence of specific components in the brine samples by comparing the mass spectra with those in the mass spectrometry library.

Identifying and validating all the components in the brine samples are very time consuming, and it is not mandatory by quality control. One option to resolve this problem is to study the chromatographic fingerprints without determining all the components in each brine sample (Ding, Ni, & Kokot, 2015; Pripdeevech & Machan, 2011; Wan, Stevenson, Chen, & Melton, 1999; Xia, Mei, Yu, & Li, 2017). The fingerprint technique, which characterizes the integral and local features of the brine samples, can be used to make comprehensive quality assessments of southern stinky tofu. Due to the highly complex GC‐MS datasets obtained from brines, chemometric techniques have become essential to analyze the chemical variability and to detect slightly and almost imperceptible composition changes (Arisseto, Vicente, Furlani, Pereira, & de Figueiredo Toledo, 2013; Li, Cai, & Shao, 2015; Lv et al., 2015; Zhang et al., 2017). Therefore, in this study, the fingerprints of organic volatile flavor compounds in the brine samples of southern stinky tofu from five manufacturers were studied using HS‐SPME/GC‐MS with the aid of chemometric methods. The fingerprints were obtained by HS‐SPME/GC‐MS and analyzed with the time shift alignment method, Shannon entropy, correlation coefficient, and principal component analysis.

## 2 EXPERIMENTAL

### 2.1 Materials

Brine samples manufactured at five production sites, referred to as Cheng, Huo, Wang, Bai, and Luo, respectively, were analyzed.

Two kinds of SPME fibers with different coatings were purchased from Supelco Inc. (Bellefonte, PA, USA), namely carboxen/polydimethylsiloxane (Carboxen/PDMS, 75 μm in thickness, black) and polyella (85 μm in thickness, white). They were preconditioned prior to the analysis in the injection port of GC according to the instructions suggested by the manufacturer.

### 2.2 Headspace solid‐phase microextraction/gas chromatography–mass spectrometry

The brine sample (5 ml) and a magnetic stir bar were placed in a 15‐ml vial. Before the insertion of SPME fiber, the vial was sealed with one Teflon cover and equilibrated for 20 min in a 60°C water bath. After that, the fiber was exposed in the upper space of the sealed vial to extract compounds for 40 min.

A GC‐MS system (QP2010 Ultra; Shimadzu, Kyoto, Japan) equipped with an Rtx‐WAX capillary column (30 m × 250 μm i.d. ×0.25 μm; Restek, Bellefonte, PA, USA) was employed. In the experiment, the electron impact ionization was tuned at 70 eV and helium (99.999%, BOC) was used as carrier gas with an average linear velocity of 1.0 ml/min. The temperatures of the GC injector and the ion sources were 250°C and 200°C, respectively. The mass range of the MS detector was from 45 to 450 m.u. The oven temperature was initially at 45°C for 2 min; then increased at 5°C/min to 150°C, which was held for 2 min; and finally raised to 290°C at 15°C/min, which was held for 10 min. The injection port was in splitless mode.

**Figure 1:**
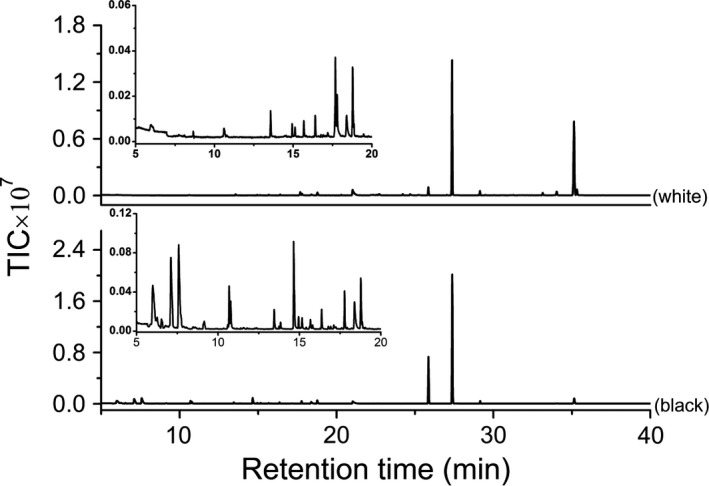
Chromatographic fingerprint of the volatile components in Huo sample by white polyella (up) and black Carboxen/PDMS (down) extraction, respectively. The figures in the top right show enlarged TICs in 5–20 min

Take the analysis of Huo sample as an example. Figure 1 shows the chromatographic fingerprints of the volatile components in the brine sample by white polyella (up) and black Carboxen/PDMS (down) extraction, respectively. As shown in the figure, the total ion chromatograms (TICs) were composed of overlapping peaks, indicating the complexity of the constituents in the extracts. Moreover, as shown in the top right, a lot of small peaks can be found in the chromatograms, forming the chromatographic fingerprints. Analysis of the other four samples shows similar results.

### 2.3 Data analysis

Fingerprints were analyzed with different chemometric methods, such as the time shift alignment method, Shannon entropy (Gong, Liang, Xie, & Chau, 2003), correlation coefficient (Keyfi & Varasteh, 2016), and principal component analysis (Li, Du, Cai, & Shao, 2012; Poole & Poole, 1995; Spínola, Perestrelo, Câmara, & Castilho, 2015). Time shifts can be accurately aligned by correlation optimized warping (COW) method (Coutinho et al., 2016; van Nederkassel, Daszykowski, Eilers, & Heyden, 2006; Tomasi, Berg, & Andersson, 2004). All calculations were carried out in MATLAB.

## 3 RESULTS AND DISCUSSION

### 3.1 Time shift correction

**Figure 2:**
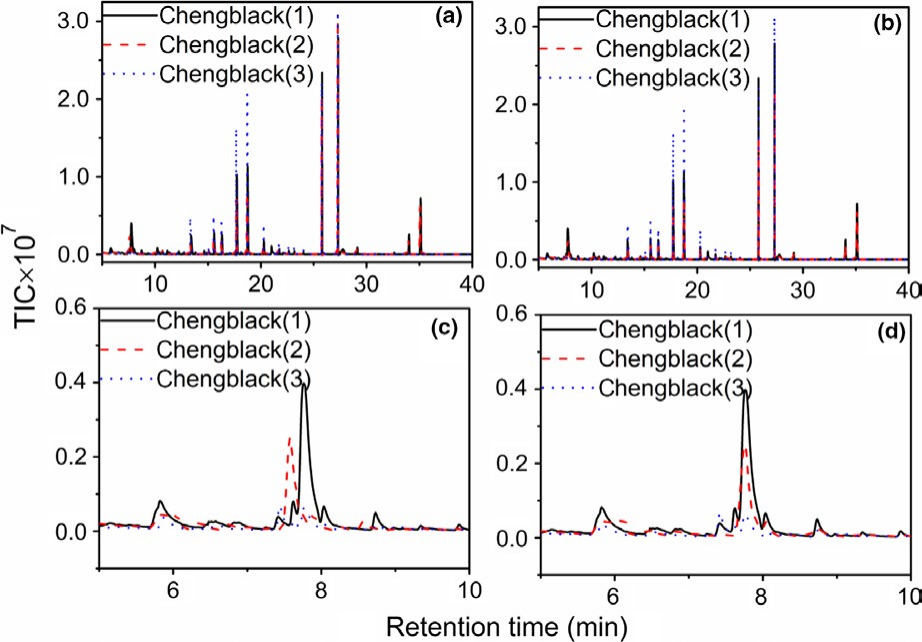
(a–d) is the TICs, enlarged TICs of Cheng sample (black Carboxen/PDMS extraction) before and after time shift correction

For the analysis of the brine samples, it might be difficult to separate the analytes from the interferences with good resolution. Figure 2a depicts the chromatographic profile of Cheng sample by black Carboxen/PDMS extraction, while Figure 2c shows the enlarged chromatographic fingerprints in 5–10 min. Apparently, the run‐to‐run retention time shift can be clearly observed, and it cannot be analyzed directly with the complex chromatographic fingerprints. In the paper, the time shifts were aligned by COW method, and the aligned chromatograms and enlarged TICs were shown in Figure 2b,d, respectively. As shown in the figures, the time shifts in the samples can be accurately corrected with the time shift alignment method despite unexpected interferences.

Moreover, the interferences of peak shifts are also serious for the chromatograms of the other four brine samples. However, with the COW method, similar results can be obtained for the analysis of them, and the run‐to‐run retention time shifts can be accurately corrected, too. Therefore, the corrected fingerprints can be used for further discussion.

### 3.2 Shannon entropy

It is very important to reasonably evaluate whether a chromatographic fingerprint carries enough information. In the work, the fingerprint information was evaluated by Shannon entropy, as shown in Table 1. Each Shannon entropy was averaged from measurements of three samples. The Shannon entropy of Wang sample by white polyella extraction is the largest, showing the highest degree of separation and component information. In combination with the normalization method, the total peak number in the fingerprint of Wang sample by white polyella extraction is the largest, verifying the accuracy of the Shannon entropy method. Besides, all the values of Shannon entropy are >1, showing that the fingerprints of all the five brine samples carry a certain amount of information and therefore have some research value. It is a simple and reasonable evaluation method to evaluate the information of chromatographic fingerprints by Shannon entropy.

**Table 1:** Shannon entropy of the five brine samples

Shannon entropy	Cheng	Huo	Wang	Bai	Luo
White polyella extraction	2.2521	4.9032	9.5040	3.0056	8.8971
Black Carboxen/PDMS extraction	1.7449	2.7983	3.5075	2.5112	3.0574

### 3.3 Correlation coefficient

The similarities and differences in the fingerprints were investigated by correlation coefficient, as shown in Table 2. Each correlation coefficient was averaged from measurements of three samples. The correlation coefficients of Wang and Luo samples are >0.5810, proving that there are similarities of raw materials and process parameters between them. The other correlation coefficients are <0.3000, indicating little similarity of raw materials and process parameters among the other three samples.

**Table 2:** The correlation coefficients and *p* values of the five brine samples

	Huo	Wang	Bai	Luo
White polyella extraction				
Cheng	0.2859 (0.00)^a^	0.0955 (0.00)	0.1973 (0.00)	0.0017 (0.47)
Huo		0.0726 (0.00)	0.2060 (0.00)	0.0077 (0.03)
Wang			0.0394 (0.00)	0.5810 (0.00)
Bai				0.0017 (0.05)
Black Carboxen/PDMS extraction				
Cheng	0.0530 (0.00)	0.1205 (0.00)	0.1736 (0.00)	0.0865 (0.00)
Huo		0.2010 (0.00)	0.2180 (0.00)	0.2777 (0.00)
Wang			0.2171 (0.00)	0.7064 (0.00)
Bai				0.1578 (0.00)

a *p* value is listed in parentheses.

### 3.4 Principal component analysis

**Figure 3:**
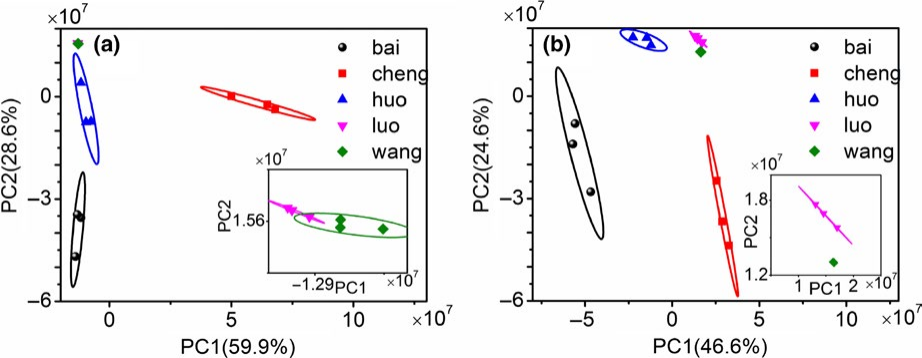
Principal component analysis of the five brine samples by white polyella extraction (a) and black Carboxen/PDMS extraction (b), respectively

In order to discriminate the samples from the five manufacturers, principal component analysis was performed. Figure 3 shows the classification effect of the five datasets. From the explained variances labeled in the axes, the first two scores (PC1 and PC2) are sufficient for analysis. In general, the five brine samples can be distinguished from each other by principal component analysis. However, for the dataset measured with white polyella extraction, as shown in Figure 3a, the data of Luo and Wang merge together. Figure 3b shows a better result, but groups Luo and Wang are still close to each other. The results show that there is a similarity of raw materials and process parameters between Luo and Wang samples, which is consistent with the conclusion of section 3.3.

Furthermore, the identified compounds were determined by comprising the mass spectra with those in the mass spectrometry library. The match ratios are above 80%, giving the positive answer of the existence of the compound. The types of compounds identified are similar to those obtained from fermented stinky tofu (Liu et al., 2009). A total of 24 typical volatile compounds were identified in Luo sample by comparing the mass spectra with those in the mass spectrometry library, while 23 typical volatile compounds were identified in Wang sample. There are 9 same common components in the two samples, including ethanol, acetic acid, propionic acid, butyric acid, phenol, 4‐methylphenol, diethylene glycol ethyl ether, indole, and 3‐methylindole, determining the great similarity between the fingerprints of the two samples. From Table 3, which lists the common components in the five samples, it can be found that indole and 3‐methylindole, with very strong unpleasant odors, typical volatile flavor compounds of southern stinky tofu brine, exist in all the brine samples. Phenol and 4‐methylphenol, as both flavor compounds and bactericides, can also be found in all the five brine samples.

**Table 3:** The common components in the five brine samples

Category	Chemical name	White polyella extraction	Black Carboxen/PDMS extraction	Match ratio (%)	Retention time (min)
Alcohols	Ethanol^b,c,e^	√^a^	√	95	1.322
Acids	Acetic acid^a,c,d,e^	√	√	97	13.969
	Propionic acid^a,c,e^	√	√	90	15.683
	Butyric acid^b,c,d,e^	√	√	97	17.82
	Pentanoic acid^b,c,d^	√	√	98	20.327
	3‐Methylbutanoic acid^a,c,d^	√	√	91	19.136
Ether	Dimethyl disulfide^a,b,d,e^		√	97	4.59
	Diethylene glycol ethyl ether^b,c,e^	√		97	8.21
Phenols	Phenol^a,b,c,d,e^	√	√	97	25.81
	4‐Methylphenol^a,b,c,d,e^	√	√	98	27.319
	4‐Ethylphenol^a,d,e^	√	√	97	29.1
Heterocycles	Indole^a,b,c,d,e^	√	√	97	33.97
	3‐Methylindole^a,b,c,d,e^	√	√	98	35.07

^a‐e^The components were detected from the brine samples of Cheng, Huo, Wang, Bai, and Luo, respectively.

a The component was detected by the SPME fiber.

The different compounds were summarized in Table 4, which may be due to the differences between the manufacturing processes. The different compounds are esters, alcohols, sulfides, organic acids, aldehydes, and ketones. The ester compounds can impart bines with fruity notes and make the odor of brine lifting and diffusive. The formations of alcohol compounds may be due to the fermentation of carbohydrates from soybean during the ripening step, when the sulfide compounds arise from the degradation of amino acids containing sulfur. The ester, alcohol, aldehyde, and ketone components may give the different brands of the southern stinky tofu brines different fruity and sweet odors. However, the aroma intensities of indole and sulfides exceed their aroma intensities, and they give the brine its very strong unpleasant odor.

**Table 4:** The different components in the five brine samples

Brand	Chemical name	White polyella extraction	Black Carboxen/PDMS extraction	Match ratio (%)	Retention time (min)
Cheng	Hexanal		√	91	4.689
	2‐Pentylfuran		√	97	7.441
	Ethoxyethanol		√	97	8.21
	Octanal		√	86	9.103
	Nonanal		√	95	11.815
	3‐Methylbutyric acid	√^a^		92	18.682
	2‐Methylpentanoic acid	√	√	96	20.927
	3‐Phenylpropanol		√	93	27.273
	5‐Hydroxy‐4‐octanone	√		84	33.096
Huo	Dimethyl Sulfide		√	97	1.76
	Pyrrole		√	81	15.619
	Tetrahydropyran	√		84	17.744
	2‐Methyl octanoic acid	√		90	24.222
Wang	5‐Methyl‐3‐cycloheptanone		√	92	8.542
	3‐Ethylcyclopentanone		√	85	10.337
	Isooctyl alcohol		√	94	14.542
	Terpineol		√	88	19.389
	1, 4‐Butanediol	√		89	24.196
	Benzothiazole	√	√	86	24.703
	Diglycol	√		89	25.183
	Cedrenol		√	80	28.062
	Amyl alcohol	√	√	98	9.076
	3‐Hydroxy‐2‐butanone	√	√	94	10.589
	N‐octanol	√		96	16.52
	N‐nonanol	√		90	18.926
	Decyl alcohol	√		84	21.258
	Amyl butyrolactone	√		93	26.365
	Octanoic acid	√	√	96	27.081
	1‐Tetradecanol	√		95	29.356
	Decanoic acid	√		86	31.377
	N‐butanol	√		85	35.352
	Phenylacetic acid	√		87	37.098
	Di‐n‐butyl Phthalate	√		89	42.533
Luo	3‐Octanone		√	97	1.747
	2‐Octanone		√	92	1.8
	Dimethyl trisulfide		√	96	4.594
	3‐Octanol		√	97	4.603
	Γ‐Thiobutyrolactone		√	88	8.277
	2‐Borneol		√	96	9.091
	Cedrol		√	94	12.21
	3‐Methyl‐3‐buten‐1‐ol		√	80	13.97
	α‐cedrene		√	96	16.392
	2‐Undecanone		√	82	16.691
	Benzothiazole	√		82	20.562

a The component was detected by the SPME fiber.

## 4 CONCLUSION

The fingerprints of organic volatile flavor compounds in southern stinky tofu brine samples from five manufacturers were studied using HS‐SPME/GC‐MS with the aid of chemometric methods. The fingerprints were obtained by HS‐SPME/GC‐MS and analyzed with the time shift alignment method, Shannon entropy, correlation coefficient, and principal component analysis. The results show that the time shifts in the samples can be accurately corrected with the time shift alignment method despite unexpected interferences. The fingerprint information was evaluated by Shannon entropy, while the similarities and differences in the fingerprints were investigated by correlation coefficient. Moreover, the identification of manufacturers was achieved by principal component analysis. The predominant volatile compounds in southern stinky tofu brine were indole, 3‐methylindole, phenol, and 4‐methylphenol.

## ACKNOWLEDGEMENTS

This study was supported by National Natural Science Foundation of China (No. 31571819, 31601551, and 31671931) and the “1515 Talent Project” of Hunan Agricultural University.

## CONFLICT OF INTEREST

The authors notify that there are no conflicts of interest.

## ETHICAL STATEMENTS

This study does not involve any human or animal testing.
